# YY1 directly interacts with myocardin to repress the triad myocardin/SRF/CArG box-mediated smooth muscle gene transcription during smooth muscle phenotypic modulation

**DOI:** 10.1038/s41598-020-78544-3

**Published:** 2020-12-11

**Authors:** Jian-Pu Zheng, Xiangqin He, Fang Liu, Shuping Yin, Shichao Wu, Maozhou Yang, Jiawei Zhao, Xiaohua Dai, Hong Jiang, Luyi Yu, Qin Yin, Donghong Ju, Claire Li, Leonard Lipovich, Youming Xie, Kezhong Zhang, Hui J. Li, Jiliang Zhou, Li Li

**Affiliations:** 1 Department of Internal Medicine, Wayne State University, 421 E. Canfield Ave. #2146, Detroit, MI 48201 USA; 2 Center for Molecular Medicine and Genetics, Wayne State University, 421 E. Canfield Ave. #2146, Detroit, MI 48201 USA; 3 Cardiovascular Research Institute, Wayne State University, 421 E. Canfield Ave. #2146, Detroit, MI 48201 USA; 4 Barbara Ann Karmanos Cancer Institute, Wayne State University, Detroit, MI 48201 USA; 5 Department of Pharmacology and Toxicology, Medical College of Georgia, Augusta University, Augusta, GA 30912 USA; 6 The Institute of Translational Medicine, Nanchang University, Nanchang, 330031 Jiangxi China; 7 Bone and Joint Center, Henry Ford Hospital, Detroit, MI 48202 USA; 8 Department of Medicine, University of Massachusetts, Worcester, MA 01655 USA; 9 Present Address: College of Medicine, Mohammed Bin Rashid University of Medicine and Health Sciences, Dubai, UAE

**Keywords:** Transcription, Restenosis

## Abstract

Yin Yang 1 (YY1) regulates gene transcription in a variety of biological processes. In this study, we aim to determine the role of YY1 in vascular smooth muscle cell (VSMC) phenotypic modulation both in vivo and in vitro. Here we show that vascular injury in rodent carotid arteries induces YY1 expression along with reduced expression of smooth muscle differentiation markers in the carotids. Consistent with this finding, YY1 expression is induced in differentiated VSMCs in response to serum stimulation. To determine the underlying molecular mechanisms, we found that YY1 suppresses the transcription of CArG box-dependent SMC-specific genes including *SM22α*, *SMα-actin* and *SMMHC*. Interestingly, YY1 suppresses the transcriptional activity of the *SM22α* promoter by hindering the binding of serum response factor (SRF) to the proximal CArG box. YY1 also suppresses the transcription and the transactivation of *myocardin* (MYOCD*),* a master regulator for SMC-specific gene transcription by binding to SRF to form the MYOCD/SRF/CArG box triad (known as the ternary complex). Mechanistically, YY1 directly interacts with MYOCD to competitively displace MYOCD from SRF. This is the first evidence showing that YY1 inhibits SMC differentiation by directly targeting MYOCD. These findings provide new mechanistic insights into the regulatory mechanisms that govern SMC phenotypic modulation in the pathogenesis of vascular diseases.

## Introduction

Vascular smooth muscle cells (VSMC) differentiation, characterized by the expression of VSMC contractile proteins, is required for a variety of physiological functions including the regulation of blood pressure and blood flow distribution^1^. The regulation of VSMC differentiation is an important process for proper vascular maturation during embryogenesis and in vascular remodeling in response to environmental cues. Extensive studies have elucidated the critical roles of serum response factor (SRF) and its co-factor myocardin (Myocd) in regulating SMC phenotypic modulation in development and in the pathogenesis of vascular diseases^2–6^. SRF plays a central role in regulating the transcription of SMC-specific genes including SM22α (aka SM22, Tagln), smooth muscle α-actin (SMα-actin, aka Acta2), and smooth muscle-specific myosin heavy chain (SMMHC, aka Myh11)^7^. SRF recruits MYOCD to activate the transcription of an array of SMC contractile genes by forming the MYOCD/SRF/CArGbox triad (known as the ternary complex)^2,8^ on the CArG box, a highly conserved cis-regulatory element CC(A/T)_6_GG that can be found in the promoters of most SMC-specific genes^2,3^. Given the complexity of vascular remodeling in development and in vascular diseases, the underlying molecular mechanisms for SMC phenotypic modulation remain largely unknown.

Yin Yang-1 (YY1) belongs to the GLI-Kruppel class of zinc finger proteins that can act as a transcriptional repressor or activator depending on the cellular context^9^. YY1 has been shown to regulate gene transcription by either binding to the promoter or as a cofactor of a variety of transcription factors^9^. YY1 plays critical roles in embryonic development^10^ as well as in a variety of pathophysiological processes involved in cytokinesis, apoptosis, cell differentiation and cell cycle^9^. In the vascular system, YY1 has been shown to inhibit neointima formation in human, rabbit and rat blood vessels due to its inhibitory effect on SMC growth^11^. However, the role of YY1 in SMC differentiation is yet defined.

SM22α is a widely used SMC differentiation marker whose transcription is controlled by a proximal CArG box (also known as CArGnear) in the *SM22α* promoter^12–16^. It has been shown that YY1 binds to the CArG box in the *SM22 α* promoter and acts as a transcriptional activator in SMCs^13^, while a series of other studies found that YY1 represses the promoter activity of smooth muscle^17,18^, cardiac muscle^19^ and skeletal muscle^20^ genes. Yet, so far the underlying molecular mechanisms of YY1 in regulating SMC gene transcription remains largely unknown.

In the present study, we found that YY1 expression is induced in media SMCs of carotid arteries upon injury accompanied by the downregulation of the transcription of SMC differentiation markers. Mechanistically, YY1 represses MYOCD/SRF/CArG box-mediated gene transcription in VSMCs by competitively displacing MYOCD from SRF to repress SMC gene transcription. Our results suggest that YY1 inhibits SMC differentiation as a transcription repressor.

## Results

### Carotid injury induces YY1 expression accompanied by reduced SMC differentiation in the vessel wall

**Figure 1:**
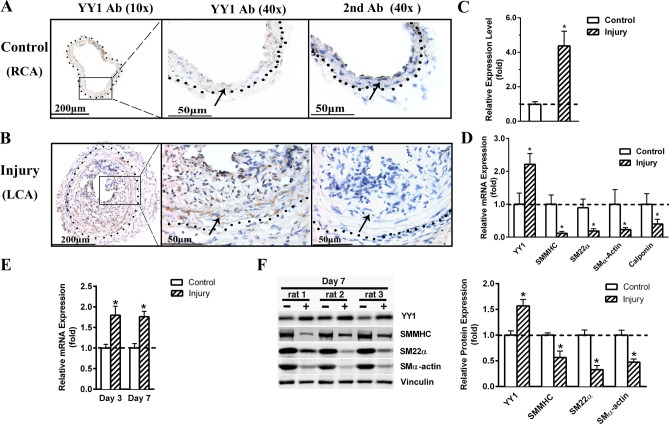
Carotid injury induces YY1 expression and inhibits SMC differentiation. (**A–D**) Mouse carotid arteries were ligated for two weeks. (**A,B**) Representative IHC images show that YY1 expression is low in the uninjured right carotid artery (Control, RCA) and injury induces YY1 expression in the neointima-media layer of the injured left carotid artery (Injury, LCA). The YY1 antigen positive signal is increased in the injury-induced neointima-media layer between the lumen and the adventitia. The black dots indicate the border between media and adventitia of the vessel wall. The adjacent section incubated with the secondary antibody (2^nd^ Ab) alone serves as the negative control. The arrow indicates the same position in the media layer SMCs on two adjacent sections. Scale bar: 200 μm (10X); 50 μm (40X). (**C**) Quantification of positive IHC signals in the neointima-media layer of the vessel wall. n = 4. (**D**) qRT-PCR assays show increased expression of YY1 and decreased expression of SMC differentiation markers in injured carotid arteries. n = 6. (**E,F**) Rat carotid arteries after balloon injury for 3 or 7 days. (**E**) qRT-PCR assays show increased expression of YY1 in response to injury. n = 6. (**F**) Western blot assays show increased expression of YY1 and decreased expression of SMC differentiation markers SMMHC, SM22α and SMα-actin in the injured carotid arteries. Quantification of band density is shown on the right. n = 3. Vinculin serves as the loading control for WB. The uncropped original blots are presented in Supplementary Figure S3. All quantification data are presented as mean ± SEM. *P < 0.05 versus control.

Previous studies showed that YY1 is highly expressed in cultured primary aortic SMCs and SMC cell lines^13,17^. Unexpectedly we found that the expression of YY1 is low in the medial layer of the control uninjured mouse right carotid artery (RCA) (Fig. 1A). However, in response to injury, YY1 expression is induced in the neointima-medial SMCs two weeks post the ligation of the left carotid artery (LCA) (Fig. 1B). Compared with the control, the expression of YY1 IHC signal in the injured carotid increased about fourfold (Fig. 1C): this result is consistent with YY1 induction in the injured rat carotid^21^. The qRT-PCR assay further revealed that the carotid injury induces YY1 mRNA level about 2 folds but represses the transcription of smooth muscle differentiation genes including *SMMHC, SM22α, and SMα-actin* and *Calponin* (Fig. 1D).

Previous studies showed that the expression of YY1 is induced in the vessel wall of the rat carotid artery following balloon injury^21,22^. Consistent with these studies, we found that YY1 expression is induced at both mRNA and protein levels 3 and 7 days after the balloon injury by qRT-PCR and Western blot (WB) assays, respectively (Fig. 1E,F). Meanwhile, the expression of smooth muscle differentiation markers including SMMHC, SM22α, and SMα-actin are downregulated by WB assays (Fig. 1F).

Taken together, we show that carotid artery injury in rodents induces YY1 expression but inhibits the expression of SMC differentiation markers in the vessel wall.

### YY1 suppresses the transcription of CArG box-dependent SMC-specific genes

**Figure 2:**
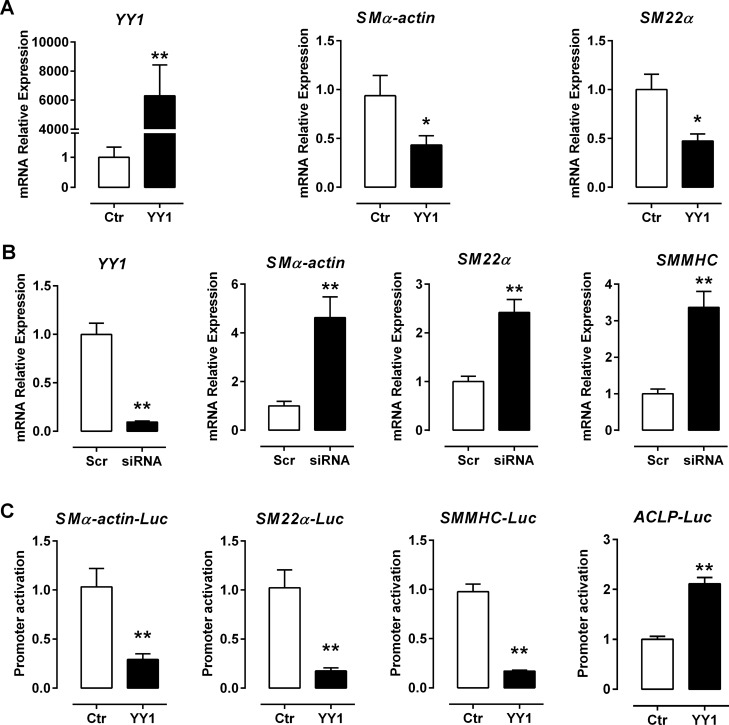
YY1 suppresses the transcription of VSMC genes encoding contractile proteins in PAC1 VSMCs. (**A**) PAC1 cells were transfected with YY1 expression plasmid (YY1) or the empty vector control (Ctr). The mRNA expression level of YY1, SMα-actin and SM22α was determined by qRT-PCR assays. (**B**) Knocking down YY1 by YY1 siRNA significantly increased the transcription of SMC-specific genes by qRT-PCR assays. Scr: the scramble siRNA as the control. (**C**) YY1 overexpression in PAC1 cells inhibited the promoter activity of SMα-actin-Luc, SM22α-Luc, SMMHC-Luc, but not ACLP-Luc by the luciferase assay. Values are mean ± SEM (n = 3) *P < 0.05, **P < 0.01 versus the control (Ctr).

To determine the molecular mechanisms whereby YY1 inhibits SMC differentiation, we examined the effect of YY1 on the transcription of SMC differentiation markers in PAC1 cells, a pulmonary artery-derived smooth muscle cell line that is easy to grow without losing SMC markers and has thus been widely used to study the molecular mechanisms of SMC differentiation^23–28^. qRT-PCR assays show that transfection of mammalian YY1 expression plasmid significantly increases YY1 expression but decreases the transcription of SMC-specific genes such as *SMα-actin* and *SM22α* (Fig. 2A).

To confirm the inhibitory role of YY1 on SMC gene transcription, we examined the effect of knocking down YY1 expression on the transcription of SMC differentiation marker genes. Using siRNA specifically against *YY1*, 90% of endogenous *YY1* mRNA was depleted. As a result, knocking down *YY1* in SMCs increases the transcription of *SMα-actin, SM22α* and *SMMHC* genes by qRT-PCR assays (Fig. 2B). The expression level of YY1 protein after overexpressing or knocking down in SMCs was validated by the WB assay (Figure S1). Taken together, data from both gain-of-function and loss-of-function assays of YY1 support the role of YY1 as a repressor of SMC-specific gene transcription.

To determine the mechanism underlying YY1-mediated effects on the transcription of SMC-specific genes, we performed luciferase reporter assays in PAC1 cells transfected with the *YY1* expression plasmid. We observed that YY1 overexpression markedly inhibits the promoter activity of *SMα-actin, SM22α* and *SMMHC,* all of which contain the CArG box in their promoters (Fig. 2C). In contrast, YY1 overexpression increases the promoter activity of *ACLP* (aka Aebp1) (Fig. 2C), a non-CArG dependent SMC marker gene^29^. Taken together, these results suggest that YY1 suppresses CArG box-dependent SMC-specific gene transcription.

### YY1 inhibits SRF binding to the proximal CArG box in the SM22α promoter

**Figure 3:**
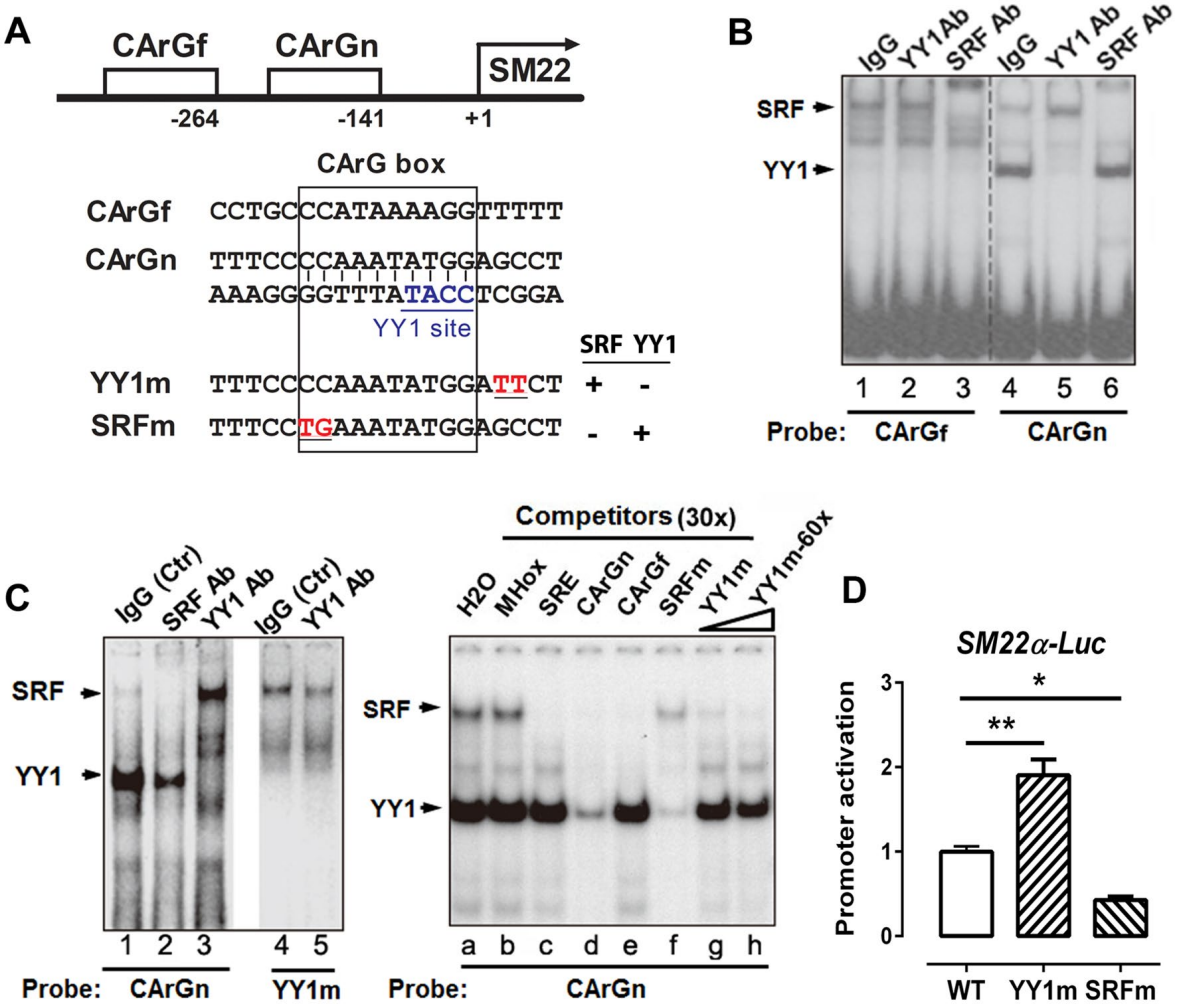
Characterization of YY1 binding to the proximal CArG box (CArGn) of the SM22α promoter in VSMCs. (**A**) The diagram to illustrate the location of CArGfar (CArGf) and CArGnear (CArGn) boxes at about 264-bp and 141-bp upstream of the transcription initiation site in the SM22α promoter, respectively. The CArGn box contains an overlapping CArG box and YY1 binding site. Mutations were generated in YY1m and SRFm oligo to selectively abolish the binding of YY1 or SRF but retain the binding of SRF or YY1 to the proximal CArG box, respectively. The mutated nucleotides are underlined and coded in red. (**B**) Gel shift assays showed the binding of SRF and YY1 to the CArG box using the nuclear extract from PAC1 cells and the indicated ^32^P labelled CArGn or CArGf oligo as the probe. The dashed line indicates noncontiguous lanes merged from the same gel. (**C**) YY1 binding site mutation selectively abolished the binding of YY1, not SRF to the YY1 mutant oligo (YY1m) (left panel). Mutation at the SRF binding site (SRFm) competed the binding of YY1, not SRF to the CArGn probe (right panel). MHox^30^ and SRE^31^ oligos served as the negative and positive control for SRF binding, respectively. (**D**) Luciferase reporters driving by the WT, YY1 binding site mutant (YY1m) or SRF binding site mutant (SRFm) of the SM22α promoter were transfected into PAC1 cells. 48 h post transfection, cells were harvested for dual luciferase assays to assess the promoter activity. The luciferase activity of the WT promoter is set at 1. Values are mean ± SEM (n = 3). **P < 0.01 versus the control (WT).

Previous studies have identified two CArG boxes (CArGfar/CArGf and CArGnear/CArGn) in the *SM22α* promoter but only the proximal CArG box (CArGn) is indispensable in controlling *SM22α* transcription^12,13^. Sequencing analyses revealed an overlapping SRF and YY1 binding site in the CArGn box, not in the CArGf box (Fig. 3A). Consistent with the sequence analysis and previous studies^13,17^, we found that YY1 binds to the CArGn box, not the CArGf box by gel shift assays using nuclear extract from PAC1 cells (Fig. 3B). It appears that the binding of SRF to the CArGn box is increased in the presence of YY1 antibody (Fig. 3B, compare lane 4 to lane 5; Fig. 3C, compare lane 1 to lane 3), suggesting that YY1 may hinder the binding of SRF to the CArG box. To confirm this possibility, the YY1 binding mutant (YY1m) oligo that selectively abolishes the binding of YY1 increases SRF binding to the CArGn box (Fig. 3C, compare lane 1 to lane 4).

Since YY1 and SRF sites are overlapping within the CArGn box (Fig. 3A), we were unable to generate a YY1 mutant that specifically abolishes YY1 binding but retains the binding of SRF by mutating the YY1 consensus sequence within the CArGn box. Literature search shows that YY1 indeed binds to the CArGn box of the SM22α promoter, but there are no evidence that mutation at the YY1 consensus sequences abolishes YY1 binding^13,17^. It is known that mutations at the flanking region of a transcription factor binding site can affect the binding of the transcription factor to its consensus core sequence. By mutating the flanking region of the YY1 consensus site we identified such a YY1 binding mutant (YY1m) in which the mutated nucleotides are underlined and coded in red (Fig. 3A). Indeed, this YY1m mutant selectively abolishes the binding of YY1 not SRF to the CArGn box (Fig. 3C, comparing lane 1 to lane 4).

Competition assays confirmed that YY1m oligo competes for the binding of SRF (but not YY1) to the CArGn box (Fig. 3C, comparing lane a, b to lane g, h). Since MHox oligo^30^ does not bind SRF, nor YY1, the MHox oligo thus serves as a background control. SRE (a classic CArG box from the c-fos promoter)^31^ serves as a positive control to compete for SRF binding to the CArGn box (Fig. 3C, comparing lane a, b to lane c). We also generated an SRF binding mutant oligo SRFm that competes for the binding of YY1 but not SRF to the CArGn box (Fig. 3C, comparing lane a, b to lane f). Taken together, these results suggest that YY1m oligo binds SRF, not YY1 while SRFm oligo binds YY1, not SRF. Thus, we have generated CArG box mutants (YY1m and SRFm) that selectively abolish the binding of either YY1 or SRF respectively to the CArGn box (Fig. 3A).

To validate the inhibitory effect of YY1 binding on the transcriptional activity of the *SM22α* promoter, we performed luciferase assays in PAC1 cells transfected with the luciferase reporter driven by the SM22α promoter, its YY1m or SRFm mutant. Compared with the wild type *SM22α* promoter (WT), mutation at the YY1 site (YY1m) increases the promoter activity about twofold. Consistent with our previous study^12^, mutation at the SRF site (SRFm) attenuates the SM22α promoter activity (Fig. 3D). These results demonstrate that the binding of YY1 to the CArGn box inhibits the transcriptional activity of the *SM22α* promoter in SMCs.

### YY1 reduces Myocd gene transcription

The results described above indicate that YY1 is a negative regulator for the transcription of SMC contractile genes, and that YY1 represses the promoter activity of SMC-specific genes in a CArG box-dependent manner. Myocardin (MYOCD) is a potent SMC-specific transcription coactivator that controls the transcription of CArG-dependent SMC genes by forming a triad with SRF/CArG box on the promoters of SMC genes^2,3^.

**Figure 4:**
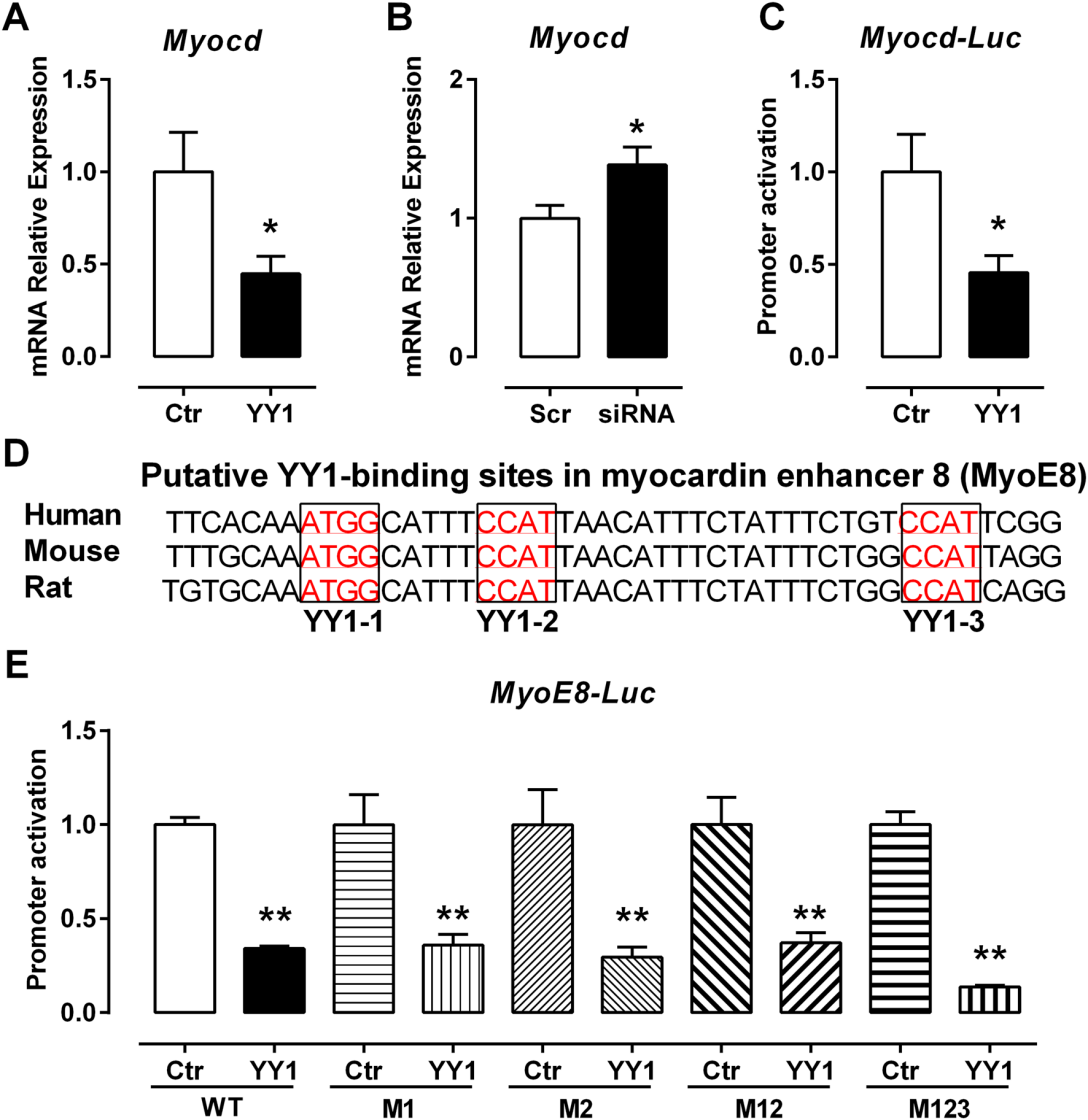
YY1 suppresses the transcription of myocardin in PAC1 cells. (**A,B**) By the qPCR assay, overexpression of YY1 downregulated the expression of *Myocd* mRNA while knockingdown YY1 by YY1 siRNA increased the expression of *Myocd* mRNA. (**C**) YY1 overexpression inhibited the Myocd promoter activity by the luciferase assay. (**D**) Three conserved putative YY1 binding sites (marked in red) were identified in the myocardin enhancer 8 (MyoE8) of human, rat and mouse. (**E**) YY1 overexpression suppressed the promoter activity of the wild type MyoE8 enhancer. Such repression could not be alleviated by mutation of each putative YY1 binding site individually or in combination. M1, M2, M12, and M123 indicate mutations of YY1 binding site YY1-1, YY1-2, both YY1-1/YY1-2, or all of three YY1 sites, respectively. Values are mean ± SEM (n = 3). *P < 0.05, **P < 0.01 versus the control (Ctr, or Scr).

To determine the role of MYOCD in YY1-mediated inhibition of SMC gene transcription, we first investigated the effect of YY1 on the transcription of *Myocd*. Following transient transfection of *YY1* expression plasmid in PAC1 cells, we found that YY1 overexpression inhibits the expression of *Myocd* mRNA by 55% (Fig. 4A). Conversely, YY1 knockdown increases the expression of *Myocd* mRNA by 38% (Fig. 4B). YY1 overexpression also suppresses the *Myocd* promoter activity by 60% (Fig. 4C), suggesting that YY1 suppresses the transcription of *Myocd* at least in part by inhibiting the *Myocd* promoter activity.

Since YY1 represses *Myocd* transcription, we next sought to determine if YY1 directly binds to the *Myocd* promoter region. We scanned for YY1 binding consensus sequence in the *Myocd* promoter by searching the public available JASPAR database, an online database for profiling transcription factor binding sequence (http://jaspar.genereg.net/). In the promoter region of *Myocd* we did not find any putative YY1 binding site. Previous study showed that the *Myocd* enhancer 8 (MyoE8), a distal upstream enhancer element, controls the transcription of *Myocd* specifically in the cardiovascular system during embryogenesis and in adulthood in mouse^32^. Therefore, we examined the mouse MyoE8 DNA sequence for YY1 binding sites. Interestingly, we found three putative YY1 binding motif sequences that are evolutionarily conserved among human, mouse and rat (Fig. 4D, Figure S2A). The top-scoring YY1 motif found in JASPAR databases resembles the known YY1 consensus sequence as indicated in a sequence logo representation^33^. These putative YY1 sites are located in the region containing multiple transcription factor binding sites including the E-box and a Foxo site in the MyoE8 enhancer^32^*.*

To determine if these putative YY1 binding sequences are functional in regulating the transcriptional activity of the MyoE8 enhancer, we mutated the three putative YY1 sites individually and in combination. After transfection of YY1 and each of the WT or mutated MyoE8-luciferase reporter in PAC1 cells, we found that YY1 overexpression decreases the transcriptional activity of the MyoE8 enhancer by luciferase assays (Fig. 4E). However, this suppression cannot be reversed by mutating these putative YY1 binding sites individually or in combination (Fig. 4E). This result suggests that YY1-mediated repression of the MyoE8 enhancer may occur through yet to be identified regulatory elements other than these putative YY1 binding sites.

### YY1 inhibits the transactivation activity of Myocd on SMC-specific gene promoters

**Figure 5:**
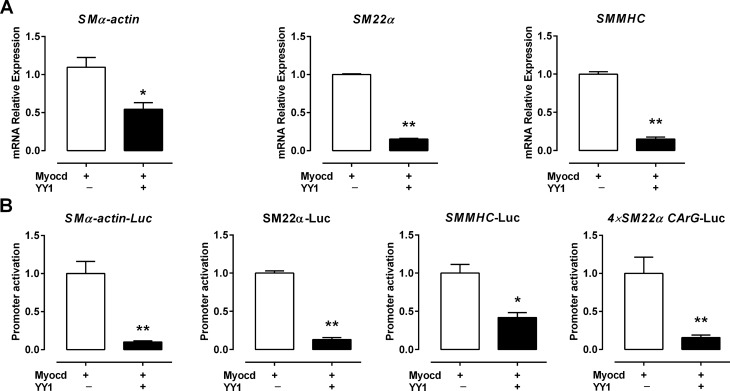
YY1 inhibits the transactivation activity of Myocd on SMC-specific gene transcription. (**A**) Mammalian expression plasmids encoding *YY1* or *Myocd* were co-transfected into 10T1/2 cells. qRT-PCR assays were performed to examine the effect of YY1 co-transfection on Myocd-induced expression of SMC-specific contractile genes such as *SMα-actin, SM22α* and *SMMHC*. (**B**) *Myocd* expression plasmid was co-transfected with luciferase reporters with or without the *YY1* expression plasmid into 10T1/2 cells as indicated. 48 h post transfection, cells were harvested for luciferase activity. Values are mean ± SEM (n = 3). The Myocd-induced SMC-specific gene expression or promoter activity was set to 1. *P < 0.05, **P < 0.01 versus the control (Ctr).

Myocd plays a critical role in regulating SMC gene transcription by binding to the SRF/CArG box complex in SMC-specific gene promoters^2,3^. To investigate whether YY1 suppresses the transactivation activity of Myocd, we examined the effect of transfecting *Myocd* on the transcription of SMC genes in 10T1/2 cells, a fibroblast cell line that has been widely used to investigate the pro-myogenic activity of Myocd due to undetectable endogenous Myocd expression^2,3^. We found that co-transfection of YY1 significantly represses *Myocd*-induced SMC gene expression by the qRT-PCR assay (Fig. 5A). Luciferase assays showed that YY1 is a potent inhibitor of Myocd-mediated transactivation of promoters of *SMα-actin, SM22α* and *SMMHC* genes (Fig. 5B). Moreover, YY1 also significantly suppresses the transactivation activity of Myocd on the 4xCArGn box reporter gene which contains four tandem repeats of CArGnear box (CArGn) of the SM22*α* promoter^12^. Taken together, these results suggest that YY1 represses SMC gene transcription by attenuating the transactivation of Myocd on the CArG box.

### YY1 directly interacts with MYOCD thereby disrupting the association of MYOCD/SRF

Extensive studies have shown that the transactivation activity of MYOCD on SMC promoters are mediated by its association with SRF^2,3^. We thus investigated the effect of YY1 on the association of MYOCD and SRF. To determine if YY1 interacts with MYOCD/SRF, we performed co-immunoprecipitation assays in PAC1 cells transfected with myc-tagged MYOCD. Nuclear extracts were incubated with anti-myc antibody and the western blot assay revealed the presence of both endogenous YY1 and SRF in the myc-MYOCD immunoprecipitants. MYOCD is known to interact with SRF. Here, we found that YY1 and MYOCD also interact in vivo (Fig. 5A).

**Figure 6:**
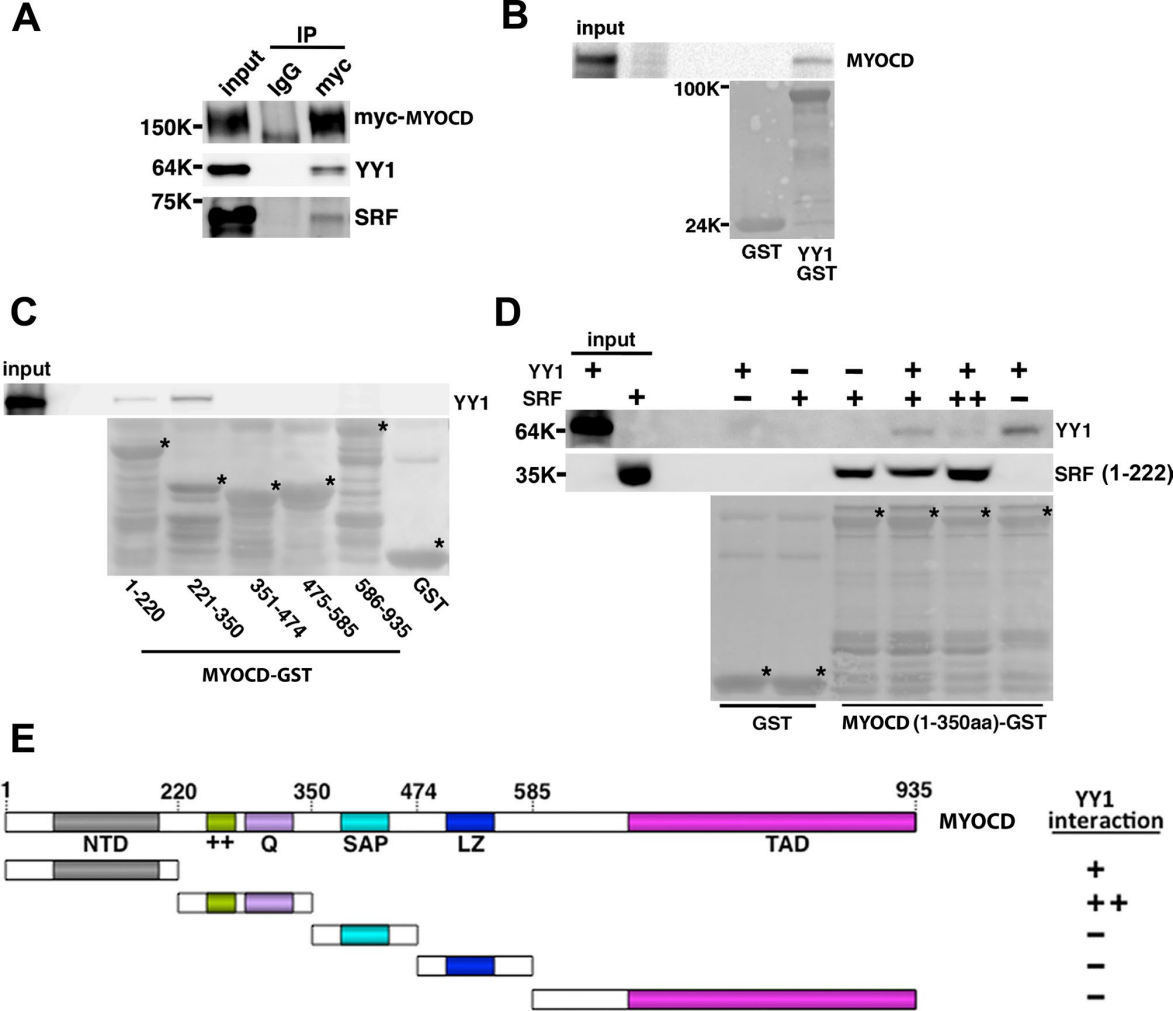
YY1 interacts with MYOCD to compete for the binding to SRF. (**A**) Expression plasmids encoding *myc-Myocd* were transfected into PAC1 cells. Nuclear protein lysates were extracted and used for co-immunoprecipitation assays using anti-myc antibody or control IgG. The presence of YY1 and SRF in immunoprecipitates were detected by Western blot using antibody against YY1 and SRF, respectively. (**B**) GST pull-down assay was performed using bacterial expressed T7 tagged full-length MYOCD to incubate with GST beads conjugated GST-YY1 or GST alone. (**C**) MYOCD-truncation GST fusion proteins (1–220 and 221–350), not the GST protein, pulled-down T7-tagged YY1. (**D**) GST and MYOCD(1–350)-GST were used to pull down YY1 and SRF (1–222). Western blot was performed using YY1 or SRF antibodies as indicated. Increased amount of SRF reduced YY1 binding to MYOCD. * indicates the GST fusion protein detected by Coomassie staining. (**E**) A schematic diagram shows domains of MYOCD required for YY1 interaction as revealed by GST pull down assays. The uncropped original western blots for panel 6A-6D are presented in Supplementary Figure S4A-S4D, respectively.

GST-pull down assays revealed that YY1 directly interacts with MYOCD (Fig. 6B). To map the interaction domains of MYOCD with YY1, we performed GST pull down assays using a series of MYOCD-GST truncation mutants that we previously generated^34^. We found that YY1 interacts with MYOCD at the region containing the basic (+ +) and glutamine-rich (Q) domain as well as the N-terminal domain (NTD) (Fig. 6C,E). Since SRF also interacts with MYOCD at the basic and Q domain region^2^, it is likely that YY1 competes with MYOCD for the binding to the common region of SRF. We thus performed a competitive GST pull-down assay using bacterial expressed MYOCD N-terminus (1-350aa), YY1 and SRF (1–222)^35^. Results show that the interaction of MYOCD and YY1 is attenuated by SRF (1–222) in a dose-dependent manner (Fig. 6D). These results suggest that YY1 competitively displaces MYOCD from SRF thereby attenuating SMC-specific gene transcription.

### YY1 is induced in response to serum stimulation and vascular injury, leading to repressing MYOCD/SRF/CArG box-mediated SMC gene transcription through multiple mechanisms

**Figure 7:**
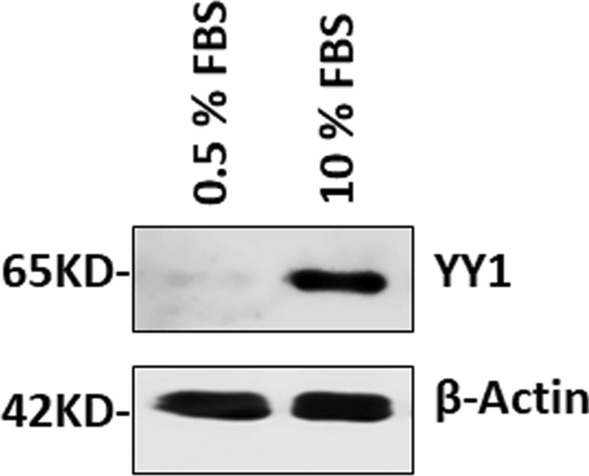
Serum stimulation in VSMCs induces YY1 expression and inhibits SRF binding to the SM22α gene promoter. Subconfluent PAC1 cells were cultured in 0.5% FBS for 48 h, followed by treatment with 10% FBS stimulation for 48 h. The western blot assay shows increased expression of YY1 in response to 10% FBS stimulation. β-actin is used as the loading control. The full-length original blots are presented in Supplementary Figure S5C.

Serum stimulation is a condition promoting SMC dedifferentiation mimicking vascular injury-induced SMC phenotypic modulation in vivo. To explore the physiological relevance of YY1-mediated repression of VSMC differentiation, we assessed the expression of YY1 in PAC1 cells under differentiation and dedifferentiation conditions by culturing PAC1 cells in low and high concentration serum medium (0.5%FBS and 10% FBS), respectively. Consistent with the in vivo results in injured carotids (Fig. 1A), we found that YY1 expression is low in differentiated PAC1 cells but is induced in PAC1 cells in response to serum stimulation (Fig. 7).

**Figure 8:**
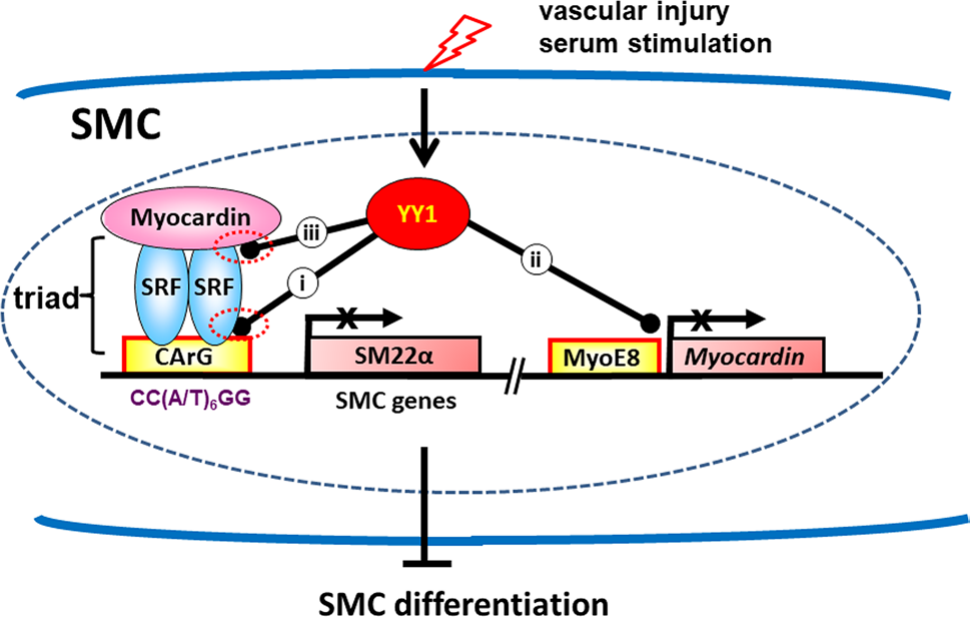
Schematic diagram of the molecular mechanisms of YY1 in suppressing the transcriptional activities of the MYOCD/SRF/CArG triad during SMC phenotypic modulation. The Myocardin/SRF/CArG box triad plays a critical role in coordinating gene transcription in SMC in response to developmental and pathogenic signals^2,4,5,40,42^. Our study here demonstrates that vascular injury or serum stimulation induces the expression of YY1 in SMCs to repress Myocardin/SRF/CArG box-mediated SMC-specific gene transcription through multiple mechanisms: (i) by inhibiting the binding of SRF to the CArG box in the SMC-specific promoter; (ii) by inhibiting the transcription activity of both the promoter and the enhancer (MyoE8) of *Myocardin* gene; and (iii) by competing with SRF for the binding to Myocardin protein. The drawing is created by contributing authors Drs. Jianpu Zheng, Jiliang Zhou and Li Li.

In summary, this study shows that YY1 is induced in VSMCs in response to vascular injury and serum stimulation to repress the triad MYOCD/SRF/CArG box-mediated SMC gene transcription via multiple mechanisms (Fig. 8): YY1 inhibits (i) the binding of SRF to the CArG box; (ii) the transcription of *Myocd* by repressing the *Myocd* promoter and the *MyoE8* enhancer; (iii) the transactivation activity of MYOCD by displacing MYOCD from SRF on the CArG box of SMC-specific gene promoters.

## Discussion

The results presented here show that YY1 is induced in VSMCs in response to stress stimuli thereby promoting SMC dedifferentiation as a potent transcription repressor both in injured vessel wall (Fig. 1) and in serum treated SMC cell line (Fig. 7). SM22α is known to be down-regulated during VSMC dedifferentiation^36,37^. Here we show that YY1 represses MYOCD/SRF/CArG box-mediated SM22α gene transcription via multiple mechanisms (Fig. 8). These findings, for the first time, reveal that YY1 directly targets MYOCD-mediated SMC-specific gene transcription thereby inhibiting VSMC differentiation.

Previous studies showed that YY1 inhibits SMC proliferation in response to vascular injury^11,21,22^. The anti-proliferation effect of YY1 is achieved by repressing the expression and function of p53-p21^Waf1/Cip1^ (key repressors of the cell cycle)^11^ and the induction of heme oxygenase-1 (an anti-proliferation protein)^22^. Therefore, YY1 has been regarded as a vascular protector to inhibit neointima formation in response to vascular injury^38^. Since YY1 inhibits SMC proliferation, there is an assumption that YY1 may stimulate SMC differentiation. Although YY1 was shown to transactivate the SM22α promoter^13^, our study here confirms the inhibitory role of YY1 in SMC differentiation as reported before^17^.

Proliferation and differentiation are generally regarded as two inversely associated biological processes. It is surprising that YY1 appears to suppress both proliferation and differentiation in SMCs. It is likely that YY1 may inhibit both processes by regulating two different key regulators. Consistent with this view, a previous study showed that YY1 inhibits proliferation by targeting p53-p21-regulated cell cycle activity and HO-1anti-proliferation function^11,22^ while our current study reveals that YY1 inhibits SMC differentiation by targeting the MYOCD/SRF/CArG box triad (Fig. 8). As the name of YY1 (Yin and Yang) implies, this study provides another example illustrating the dual regulatory roles of YY1 in different biological processes^38^.

Our results show that YY1 acts as a potent repressor for the transcription of VSMC differentiation genes using both gain-of-function and loss-of-function approaches (Fig. 2). The overlapping binding sites of SRF and YY1 in the CArG box has been shown in the promoters of several muscle genes including *SM22α*^12,13,17^, *SMMHC*^18^ and *skeletal muscle α-actin*^20^. Consistent with these studies, we find that YY1 inhibits SM22α transcription by reducing SRF binding to the CArG box in the SM22 promoter (Fig. 3). It is well established that binding of SRF to the SM22 promoter can be repressed by dedifferentiation signals such as PDGF and 10%FBS^4,39^. Mechanistically, SRF binding to the CArG box can be inhibited by other transcription factors such as Elk1^4^ and NFκB^40^. Since YY1 is induced by 10%FBS (Fig. 7), it is likely that YY1 is one of the contributors to inhibit SRF binding to SM22 promoter in response to dedifferentiation signals.

To explore molecular mechanisms underlying the inhibitory role of YY1 on SMC gene transcription, we examined the effect of YY1 on the expression and transactivation of MYOCD. The expression of *Myocd* is regulated by a variety of transcription factors and signal pathways^5^. The transcription of *Myocd* in the cardiovascular system has been shown to be controlled by the E8 enhancer that contains multiple binding sites for transcription factors such as MEF2, TEAD and Foxo^32^. Although there are three putative YY1 sites in the E8 enhancer, our data suggest that YY1 may not directly bind to these sites since simultaneous mutations of these three putative YY1 binding sites did not affect the inhibitory effect of YY1 on the enhancer (Fig. 4E). This speculation is supported by the absence of YY1 binding to the E8 region shown in the ChIP-seq analyses of the ENCODE databases (Figure S2B). Further studies should examine the possibility that YY1 may inhibit the transactivation activity of the MEF2/TEAD/Foxo-mediated regulatory network thereby repressing the E8 enhancer of *Myocd*.

Although UCSC genome browser analyses of the public available ENCODE databases did not reveal any YY1 binding to the E8 enhancer of *Myocd,* we found that there are three YY1 binding sites located in the transcription initiation region of the human myocardin (*MYOCD*) variant 1, 2, 3 and 4 (Figure S2B). Since the YY1 binding site 2 (YY1-ii) shows the strongest binding signals to the *MYOCD* mRNA variant 3 chromatin (score at 897 out of 1000) in hESC (Figure S2B), further investigation is warranted to examine the role of this YY1 binding on the transcription of *myocardin* gene in VSMCs.

The transactivation of MYOCD is modulated by a variety of transcription factors and epigenetic factors such as SRF, p65, SOX9 and HDACs/p300 either in CArG-dependent or CArG-independent manner^5,40–43^. Interestingly, the basic region (+ +) and glutamine-rich region (Q) of MYOCD are required for MYOCD interaction with SRF and/or a variety of other transcription factors^42^. Here we find that this +  + /Q domain also mediates the interaction of MYCD and YY1 (Fig. 6E): this is consistent with the competitive relationship of YY1 and SRF for their interaction with MYOCD (Fig. 6D).

It is likely that under normal condition, the expression of YY1 is inhibited to ensure SMC differentiation. Consistent with a previous study^11^, we found that YY1 is induced in the vessel wall in response to vascular injury (Fig. 1). During SMC phenotypic modulation, YY1 appears to play diverse roles that may be required to maintain vessel wall homeostasis. Further studies using SMC-selective deletion of YY1 mice will facilitate the discovery of the precise role of YY1 in SMC biology and to determine how YY1 coordinates SMC proliferation and differentiation.

In summary, YY1 is a multifunctional protein that regulates SMC phenotypic modulation in response to vascular injury. In SMCs, YY1 acts as a potent transcriptional repressor to inhibit SMC differentiation by directly disturbing the interplay of key transcriptional regulators SRF, its cofactor MYOCD and their binding to the CArG box in SMC promoters.

### Significance

VSMC phenotypic modulation plays an important role in the pathogenesis of vascular diseases including atherosclerosis, restenosis and aneurysm. It is well established that YY1 inhibits VSMC growth and intimal thickening in response to vascular injury. However, the role of YY1 in SMC differentiation remains undefined. In this study, we revealed the underlying molecular mechanisms of YY1 in suppressing MYOCD/SRF/CArG box-mediated transcriptional regulation of SMC-specific genes. This study provides the first evidence showing that YY1 regulates SMC phenotypic modulation by directly targeting MYOCD, a master regulator of SMC gene transcription. Complementary to the anti-proliferation role of YY1 in response to vascular injury, this study emphasizes the important and diverse roles of YY1 in SMC phenotypic modulation.

## Materials and methods

### Rodent models for vascular injury

The use of experimental rats and mice for arterial injury procedures were approved by the IACUC and Biosafety committees at Augusta University. The animal procedures are in accordance with the NIH guidelines (Guide for the care and use of laboratory animals).

### Mouse carotid ligation procedure and analyses

Adult C57BL6 male mice (Jackson Laboratory) at 3–4 month old were used for carotid ligation procedures as described before^44^. Briefly, the left common carotid was dissected and ligated near the carotid bifurcation into internal and external carotid artery branches. The right common carotid served as the uninjured control. Two weeks after the ligation, both the right and left carotid arteries were harvested for embedding in OCT medium (Tissue-Tek) and sectioned at 6-µm thickness. Sections from the ligature to the aortic arch of the carotid arteries were first scanned for vessel wall remodeling by H&E staining. Sections at about the same location of left and right carotid arteries were used for assessing the expression of YY1 using the YY1 antibody (Abcam #ab109228 1:100) by immunohistochemistry (IHC). The primary antibody was detected by DAB Peroxidase (HRP) Substrate Kit (Vector Laboratories, Inc. Cat. #Sk-4100). The goat anti-rabbit secondary antibody (Abcam ab6721, 1:100) without the primary antibody was used as the negative control of IHC assay. Threshold gated positive signal on IHC images was detected on the whole vessel wall. Digital images of all mouse sections were obtained using the Leica DM4000B light microscope. The antigen positive signals over the neointima-media layer between the lumen and the adventitia demarcated by the external elastin fiber of the artery were quantified using the integrative optical density (IOD) function in the Image-Pro Analyzer software (Media Cybernetics, Bethesda, USA). The results were confirmed by another researcher blind to the sources of the sections.

### Rat carotid artery balloon injury procedure and analyses

The rat carotid artery balloon injury was performed as described in our previous reports^45,46^. Briefly, male Sprague–Dawley rats (350 g; Taconic Farms, Germantown, NY) were anesthetized with xylazine 4.6 mg/kg and ketamine 70 mg/kg via intraperitoneal injection. The left common carotid artery was dissected by dull forceps to expose the carotid artery bifurcation. Blood flow cessation was achieved by arterial clamps and a small arteriotomy was made in the external carotid artery. A 2F Fogarty balloon embolectomy (Edwards) was inserted through the small cut and passed into the common carotid artery. After balloon inflation, the catheter was partially withdrawn and re-inserted 3 times. The right carotid artery served as an uninjured control. The right and left carotid arteries were harvested 3 and 7 days after injury. Total RNA was extracted with RNAqueous-Micro kit (Ambion). RNA was reverse transcribed into cDNA and gene expression was measured by qRT-PCR (Quantitative reverse transcription polymerase chain reaction). Protein extracts were prepared for Western blots assays using antibodies against YY1 (Santa Cruz, sc-281, 1: 2000), SMMHC (Biomedical Technologies Inc, BT-562, 1:2000), SMα-actin, (Sigma, AC-74, 1:10,000), SM22α (Abcam, ab10135, 1:5000), vinculin (Sigma, V4505, 1:3000). Images were acquired by ImageQuan LAS4000 Imaging Station (GE). The band densities were quantified using the ImageQuant TL software (GE).

### Cell culture and transfection

PAC1 cells (a pulmonary artery-derived SMC line^23^) and 10T1/2 cells (ATCC, #CCL-226) were maintained in Dulbecco’s modified Eagle medium (DMEM) supplemented with 10% fetal bovine serum (FBS), penicillin (100 U/ml) and streptomycin (100 g/ml), at 37 °C in a humidified 5% CO_2_ incubator. PAC1 cells were growing in 10% FBS until sub-confluency, then were washed in serum free PBS three times before switching to 0.5% FBS for 48 h as the differentiated condition. 10% FBS were then added to stimulate SMC phenotypic modulation for additional 48 h.

### Plasmids

The mammalian expression vector plasmids pCMV6-YY1 (OriGene Inc.), pcDNA3.1-Myc-Myocd^2^ were used for transient transfection in cultured cells. Site-directed mutagenesis (Stratagene Inc.) was used to generate mutation in the YY1 binding site using YY1m, 5′-CCAAATATGGATTCTGTGTGG, as the mutated primer or the SRF binding site using SRFm, 5′-TGAAATATGGAGCCTGTGTGG, as the mutated primer) in the proximal CArG box (CArGnear 5′-CCAAATATGGAGCCTGTGTGG) of the SM22α promoter^12^. The luciferase reporters driven by the SM22α promoter with YY1m or SRFm mutation were then generated. The luciferase reporters driven by the SM22α promoter and the 4xCArGnear promoters were described previously^12,41^. The luciferase reporters driven by the SMα-actin and SMMHC promoter were kindly provided by Dr. Gary Owens^36^. The luciferase reporters driven by the myocardin promoter and the myocardin enhancer (MyoE8) were kindly provided by Dr. Eric Olson^32^.

### RNA extraction and quantitative real-time PCR in tissue culture cells

Total RNA from cultured cells was extracted by the RNeasy Kit (Qiagen), and cDNA was synthesized using Superscript II reverse transcriptase (Invitrogen). Real-time PCR was performed using the Step-One Plus system (Applied Biosystems) in the presence of SYBR Green. *Gapdh* was used as the internal control. Changes in mRNA expression were expressed as fold change relative to the internal control. All gene specific PCR primers were designed to flank at least 2 exons (Table 1).

**Table 1:** Primer sequences for qPCR assays.

Gene name	Species	Sequence	PCR band (bp)
YY1	Mouse	F: 5′-GCAAGGCGATGAGATTATTAG	208
		R: 5′-TCAAGCATTCAGCACAGA	
*SM22α*	Mouse	F: 5′-CCTTCTCTGCCTCAACAT	124
		R: 5′-CACTACAATCCACTCCACTA	
*SMα-actin*	Mouse	F: 5′-GGAGTAATGGTTGGAATGG	106
		R: 5′-TGATGATGCCGTGTTCTA	
*SMMHC*	Mouse + Rat	F: 5′-AACGCCCTCAAGAGCAAACTCAGA	161
		R: 5′-TCCCGAGCGTCCATTTCTTCTTCA	
*Myocd*	Mouse + Rat	F: 5′-CAGTGAAGCAGCAAATGACTCGG	230
		R: 5′-GTCGTTGGCGTAGTGATCGAAGG	
*Gapdh*	Mouse	F: 5′-CGACTTCAACAGCAACTC	109
		R: 5′-GTAGCCGTATTCATTGTCAT	

Gene name	Species	Sequence	PCR band (bp)
*YY1*	Rat	F: 5′-GGAGACGACGACTACATC	124
		R: 5′-TCTTCTTGCCACTCTTCTT	
*SM22α*	Rat	F: 5′-AGGTGTGGCTGAAGAATG	123
		R: 5′-CCTGTTCCATCTGCTTGA	
*SMα-actin*	Rat	F: 5′-GGAGTGATGGTTGGAATG	106
		R: 5′-TGATGATGCCGTGTTCTA	
*SMMHC*	Mouse + Rat	F: 5′-AACGCCCTCAAGAGCAAACTCAGA	161
		R: 5′-TCCCGAGCGTCCATTTCTTCTTCA	
*Myocd*	Mouse + Rat	F: 5′-CAGTGAAGCAGCAAATGACTCGG	230
		R: 5′-GTCGTTGGCGTAGTGATCGAAGG	
*Gapdh*	Rat	F: 5′-ACCTGCCAAGTATGATGA	118
		R: 5′-GGAGTTGCTGTTGAAGTC	

### Western blotting

Equal amounts of proteins were subjected to electrophoresis on 4–12% Bis–Tris NuPAGE Mini-gel (Invitrogen), followed by transferring to PVDF membrane (Millipore Corporation, Billerica, MA) using XCell II™ Blot Module (Invitrogen, CA). The Spectra™ Multicolor Broad Range Protein Ladder (ThermoFisher, Catalog# 26,634) is included to estimate the molecular weight of the detected protein. The membrane was blocked with 5% non-fat milk at room temperature for 1 h and incubated with indicated primary antibodies against YY1 (Santa Cruz, #sc-7341, 1:1000, MW 65KD), and β-actin (Cell Signaling, #4967 s, 1:5000, MW 42KD) overnight at 4 °C. After washing and incubating with secondary antibody for 1 h, the membrane was subjected to enhanced chemiluminescence detection using SuperSignal West Pico Chemiluminescent Substrate (Pierce). The signals on the blot were detected and imaged by Imager *Kwik* Quant (Kindle Biosciences Inc.).

### Analyses of the JASPAR databases

Putative YY1 binding sites in the E8 enhancer of the mouse *Myocd* were identified by searching the JASPAR database that is a curated and derived from published collections of experimentally defined transcription factor binding sites for eukaryotes (http://jaspar.binf.ku.dk/, 2019 year). Three ATGG or CCAT elements that match the consensus binding sequence for YY1 binding are identified. Note: the format of the input FASTA (IUPAC) sequence of human/rat/mouse myocardin E8 enhancer must include “//” at the end of the sequence as shown below:

- 1 tgcagtaaaa aacaaataga acatttggat gaagccaggt ctgctacaaa cgaaaacaat
- 61 ttttttctct tcccttgaat gtttttctag tgactccttc tggaatgtta ttttgaactt
- 121 gcaaaggttc ttaaaaaaaa aacaaacaaa ctgtgtattt cttttaagca tcccctgaga
- 181 aagtcagatt taaacttagc aaaagcactg gctttttaaa agcactttct taaaatagtg
- 241 gctgtcaatc ttggtgggaa atctttaggt gcccctaatg gccagaaata gaaatgttaa
- 301 tggaaatgcc atttgcaaag acagtggtaa gggacccagt cagaa //

### Analyses of ENCODE ChIP-seq data using UCSC genome browser

In UCSC genome browser (https://genome.ucsc.edu/, 2019 year) on Human Feb. 2009 (GRCh37/hg19) Assembly, YY1 binding to the *MYOCD* gene can be visualized and displayed through following steps: 1) click to open the ENCODE Regulation (show tab), 2) click Txn Factor ChIP (Transcription Factor ChIP-seq Clusters (161 factors) from ENCODE with Factor book Motifs) (pack tab), 3) submit filter YY1 to specifically display YY1 binding sites in the *MYOCD* locus, and 4) right click each YY1 binding site to display the YY1 ENCODE Cluster score.

### Luciferase assay

PAC1 cells were plated one day before transient transfection. Subconfluent cells were co-transfected with 50 ng of indicated SMC promoter luciferase reporter, mammalian expression plasmids and 1 ng CMV-Renilla luciferase reporter (Promega) using Lipofectamine and Plus transfection agents (Invitrogen). 24 h post transfection, the luciferase activity was determined using the Dual-Luciferase Assay System kit (Promega, Madison, WI). Relative luciferase activity was presented as the fold change after normalization with the renilla luciferase reporter activity as the internal control for transfection efficiency.

### YY1 silencing by small interfering RNA

YY1 silencing was achieved by using Dicer-Substrate siRNA duplexes (IDT). PAC1 cells were transfected with 100 nM YY1 siRNA (siYY1) duplex (5′-AGCAAACUUCUUAUUACAACCGUCGAA-3′) or scrambled siRNA (scr) duplex, the universal negative control, 5′-CGACGGUUGUAAUAAGAAGUUUGCT-3′) at 60% confluence using DharmaFECT3 transfection reagent (Dharmacon). 48 h after transfection, total RNAs were isolated for qPCR assays and total proteins were extracted for Western blotting assays.

### Electrophoretic mobility shift assay (EMSA)

^32^P-labeled oligos containing the CArGn box, CArGf box or the YY1m mutation from the SM22α promoter were used as the probe in the gel shifting assay (EMSA) as described previously^12,31^. Briefly, nuclear extracts were prepared from subconflurent PAC1 cells cultured in 10% FBS. 10 µg of nuclear extract were mixed with 1 µg poly [dI-dC] in presence or absence of 1 µg antibodies against SRF (Santa Cruz, #sc-335x), YY1 (Santa Cruz, #sc-281x) or the IgG control to a final volume of 25 µl. For competition assays, the indicated excess of unlabeled oligonucleotides was added to the reaction. After 30 min incubation on ice, equal amount of ^32^P-labeled probe was added to the reaction and incubated on ice for additional 30 min. The reaction mixtures were then separated on a 5% native PAGE and X-ray film was used to detect the signal.

### Co-immunoprecipitation (Co-IP)

Co-IP assays were carried out as described previously^47,48^. Briefly, PAC1 cells were co-transfected with expression plasmids encoding myc-tagged myocardin and YY1 as indicated. 48 h after transfection, nuclear protein was harvested and Co-IP assays were performed using the nuclear complex Co-IP kit as described by the manufacturer (Active Motif, cat. #: 54,001). One hundred micrograms of nuclear protein extracts were incubated with 50 μl of anti-Myc beads (Sigma, cat. #: E6654) or 3 μg control rabbit IgG in 500 μl low salt IP buffer (Active Motif) overnight at 4 °C. Fifty microliters of anti-rabbit IgG beads (True Blot, cat. #: 00–8800-25) were added to control IgG sample for an additional 1 h with rocking and then immobilized complexes from both anti-myc and anti-rabbit IgG beads were washed six times with the low salt IP buffer with or without BSA. The immunoprecipitated proteins were then mixed with 45 μl 2 × SDS sample buffer and analyzed by Western blot as indicated. Images were acquired by ImageQuan LAS4000 Imaging Station (GE).

### GST pull-down assays

Bacterially expressed MYOCD, MYOCD truncation mutants, SRF(1-222aa)^35^ and YY1 proteins in the pGEX-4T vectors (Stratagene) or the T7 tagged pET28 vector (Novagen) were used to generate GST or T7 fusion proteins as previously described^47^. Briefly, MYOCD-GST fusion protein was incubated for 1 h with a 50% suspension of glutathione-agarose beads (Amersham Biosciences) in PBS. Fusion proteins bound to the beads were resuspended and incubated for 2 h with T7-SRF(1-222aa) and/or T7-YY1 fusion proteins in a total volume of 1 ml of binding buffer (PBS containing 1% Triton X-100, 1 mg/ml BSA with protease inhibitors). After the beads were washed 3 times in 1 ml of washing buffer (PBS containing 1% Triton X-100), the bound proteins were eluted by heating at 95 °C for 5 min in SDS sample buffer. The eluted GST and T7 fusion proteins were resolved on SDS-PAGE and detected by Coomassie blue staining or by Western blot using anti-T7 tag antibodies (Novagen, #69,522–3, 1:10,000) or SRF (Santa Cruz, #sc-335, 1:3000). Images were taken by ImageQuan LAS4000 Imaging Station (GE).

### Statistical analysis

Statistical analysis was performed using the two-tailed Student’s t-test with the Graphpad Prism software (GraphPad Software Inc., San Diego, CA, USA). All data were expressed as mean ± SEM. The numbers of experimental repeats are indicated in figure legends. A *P* value less than 0.05 was considered significant.

## Supplementary information

Supplementary Figures.

## Data Availability

All of original data are available upon request.
