# Supplementary material for: YY1 directly interacts with myocardin to repress the triad myocardin/SRF/CArG box-mediated smooth muscle gene transcription during smooth muscle phenotypic modulation
Source: Sci Rep. 2020 Dec 11;10:21781. doi: 10.1038/s41598-020-78544-3 (PMC7732823; doi:10.1038/s41598-020-78544-3)
Supplement: Supplementary file 1 — Supplementary Figures. [file 41598_2020_78544_MOESM1_ESM.pdf]

YY1 directly interacts with myocardin to repress the triad myocardin/SRF/CArG box-mediated smooth muscle gene transcription during smooth muscle phenotypic modulation

Jian-Pu Zheng[1#], Xiangqin He[5,6#], Fang Liu[5], Shuping Yin[1], Shichao Wu[1], Maozhou Yang[7], Jiawei Zhao[1]，Xiaohua Dai[1], Hong Jiang[1], Luyi Yu[5], Qin Yin[5], Donghong Ju[1,2,4], Claire Li[2], Leonard Lipovich[2+], Youming Xie[4], Kezhong Zhang[2], Hui J. Li[8], Jiliang Zhou[5*] and Li Li[1,2,3*]

[1]Department of Internal Medicine, [2]Center for Molecular Medicine and Genetics, [3]Cardiovascular Research Institute, [4]Barbara Ann Karmanos Cancer Institute, Wayne State University, Detroit MI 48201; [5]Department of Pharmacology and Toxicology, Medical College of Georgia, Augusta University, Augusta, GA 30912; [6]The Institute of Translational medicine，Nanchang University, Nanchang, Jiangxi，China 330031;
[7]Bone and Joint Center, Henry Ford Hospital, Detroit, MI 48202;
[8]Department of Medicine, University of Massachusetts, Worcester, MA, 01655.

Running Title: YY1 interacts with MYOCD to repress SMC gene transcription

# These authors contributed equally to this work.
+ The current contact for Dr. Lipovich: College of Medicine, Mohammed Bin Rashid University of Medicine and Health Sciences, Dubai, UAE. leonard.lipovich@mbru.ac.ae

* Correspondence and requests for materials should be addressed to L.L. (lili@med.wayne.edu) or J.Z. (jizhou@augusta.edu).

Li Li, Ph.D.
Department of Internal Medicine
Center for Molecular Medicine & Genetics
Cardiovascular Research Institute
Wayne State University
421 E. Canfield Ave. #2146
Detroit MI 48201
(313) 577-8749 (office)
lili@med.wayne.edu

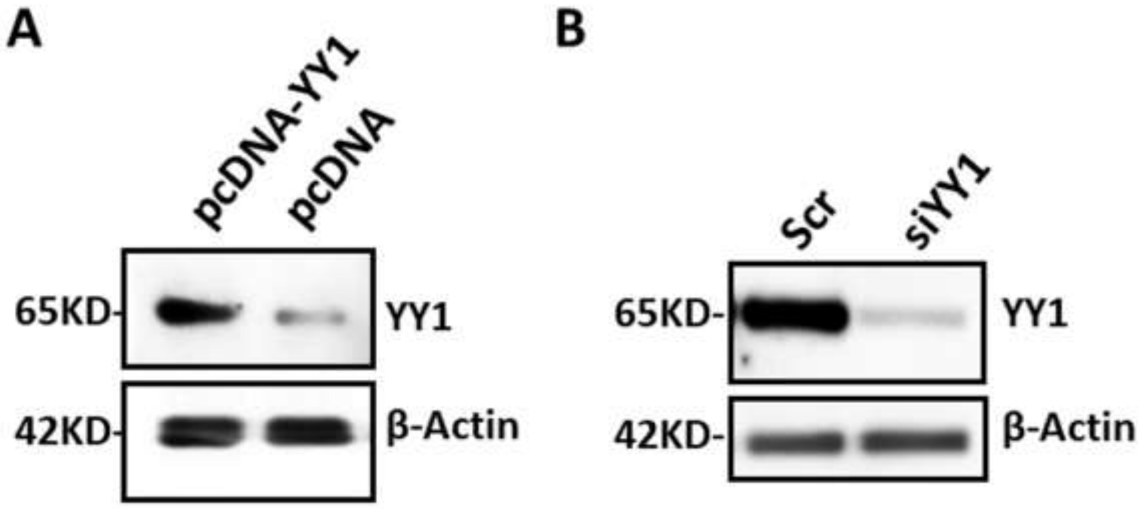

Figure S1. Validation of overexpression and knocking down YY1 in PAC1 cells by the western blot assay. (A) Expression of YY1 protein is significantly increased in PAC1 cells transfected with YY1 expression plasmid comparing to the empty vector control pcDNA. **(B)** Expression of YY1 protein is significantly reduced in PAC1 cells transfected with YY1 siRNA *(*siYY1) compared to the scramble siRNA control (Scr). β-Actin is used as the loading control. Full-length original western blots for Figure S1A & S1B are presented in Supplementary Figure S5A & S5B, respectively.

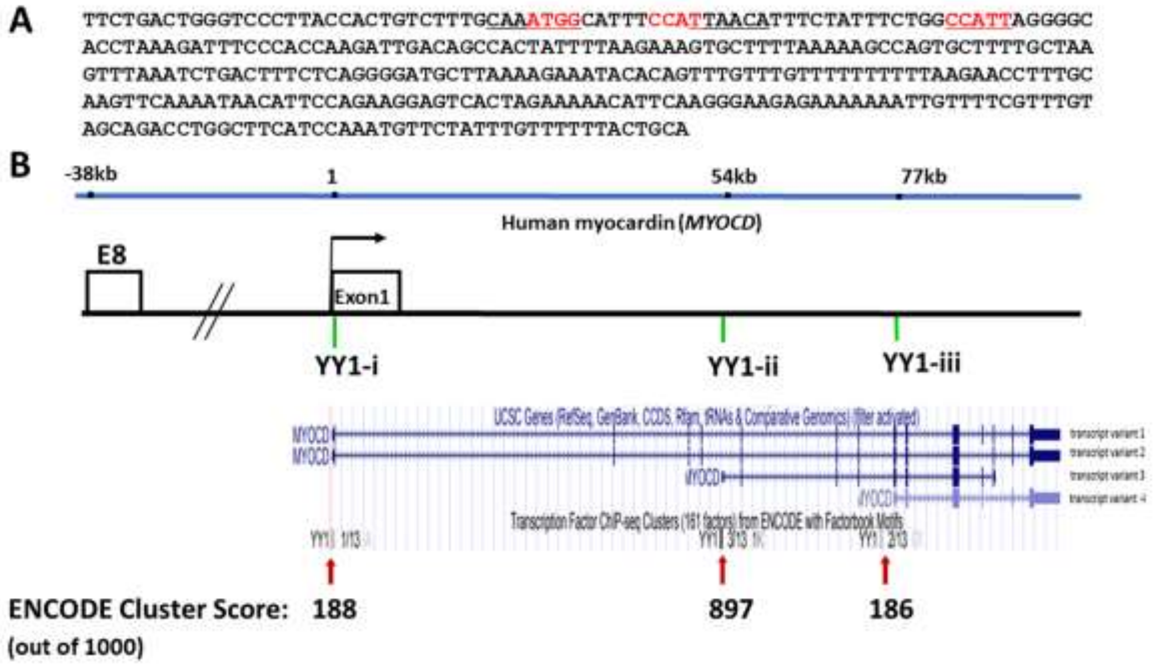

Figure S2. JASPAR and ENCODE databases identified putative YY1 binding sites in *myocardin* gene (A) JASPAR analysis revealed three putative YY1 binding sites (in red letter) in the 345-bp DNA sequence of the mouse myocardin E8 enhancer (MyoE8) originally reported by Creemers et al (PMID: 17021041). (**B**) Analyses of the ENCODE databases using the UCSC genome browser identified three YY1 binding sites at the transcription initiation region of transcript variant 1 and 2 (YY1-i), variant 3 (YY1-ii) or variant 4 (YY1-iii) of the human *MYOCD* gene respectively. The cluster scores in the ChIP-seq peaks of the YY1 binding to the chromatin are 188, 897 and 186 out of 1000 respectively. However, this ChIP-seq analysis failed to identify any YY1 binding sites in the human myocardin (*MYOCD*) enhancer (E8).

Figure 1F
Uncropped Western blots

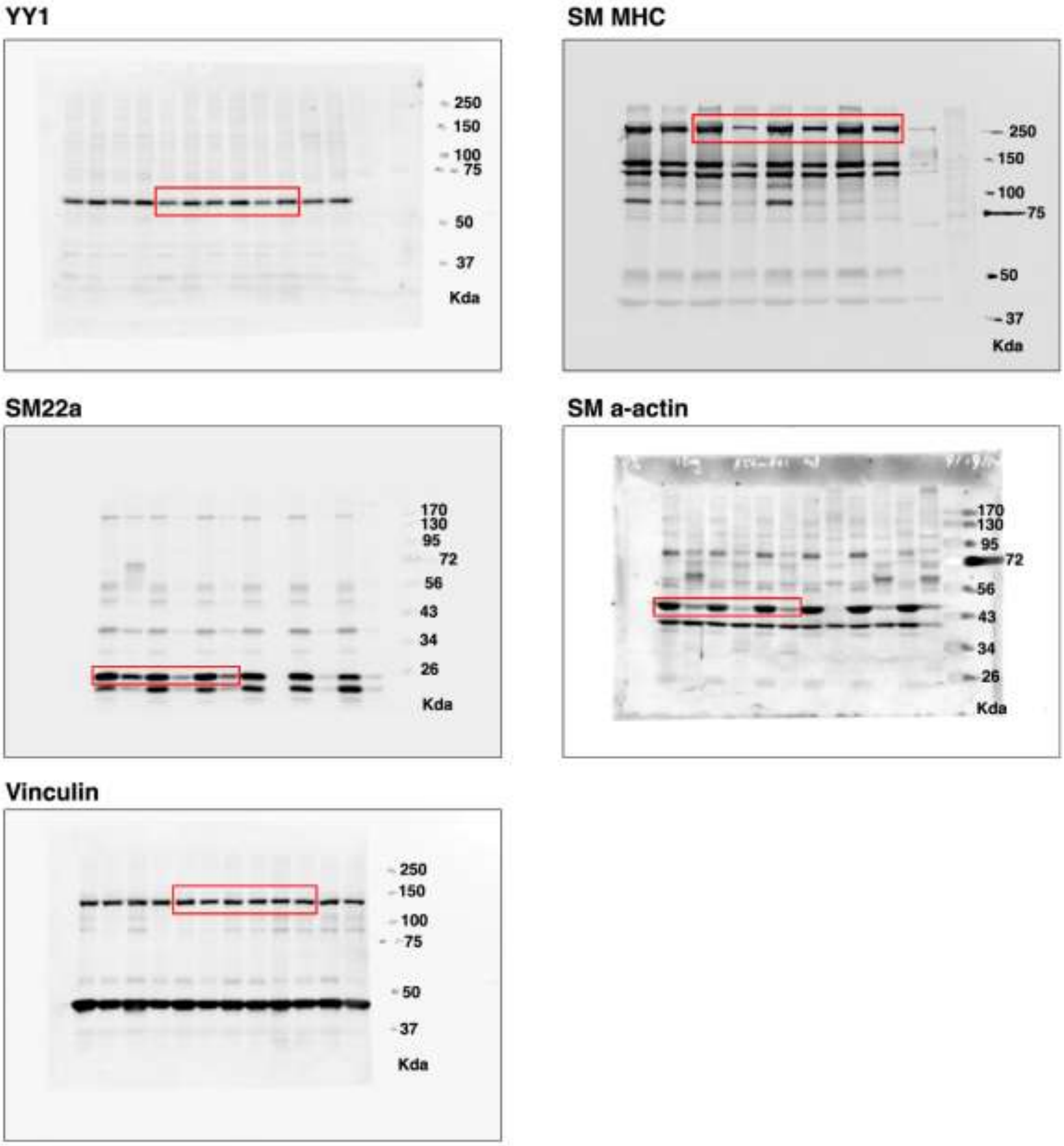

Figure S3. Uncropped Western blot images. Boxed bands on the original images by the red lines were cropped for Figure 1F. The information of each lane in the red box is the same as the corresponding lane shown in Figure 1F. All other lanes are not shown in Figure 1F.

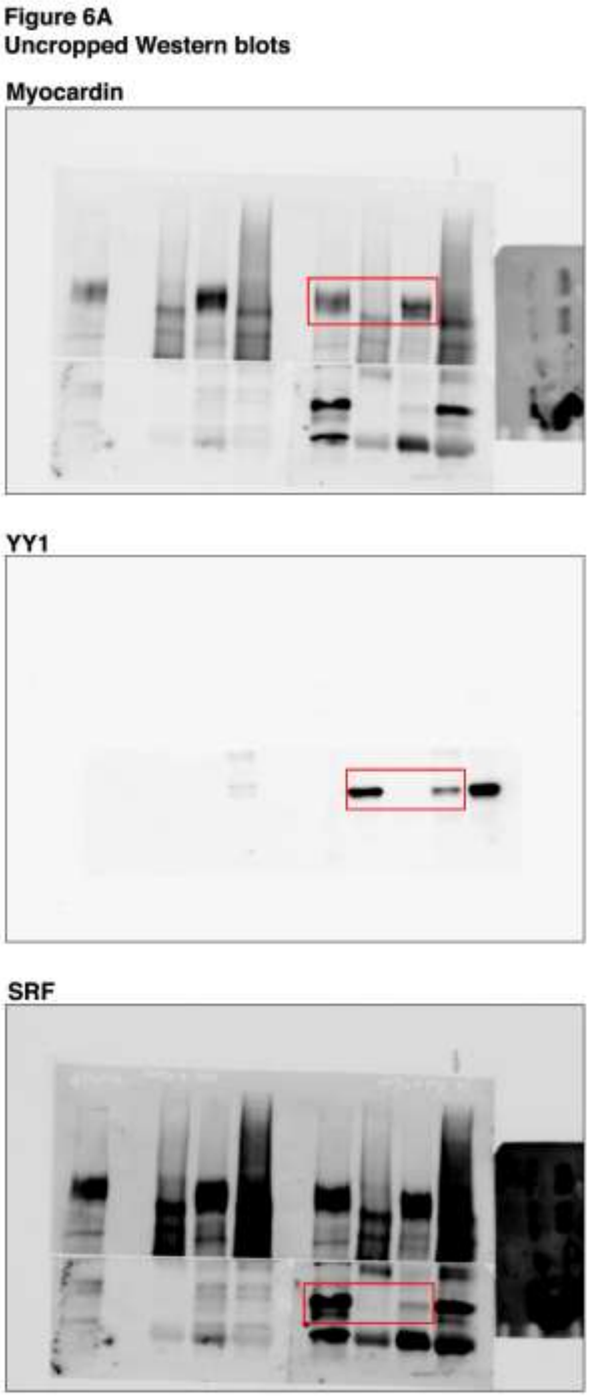

Figure S4A. Uncropped Western blot images for Fig 6A. Boxed bands on the original images by the red lines were cropped for Figure 6A. The information of each lane in the red box is the same as the corresponding lane shown in Figure 6A. All other lanes are not shown in Figure 6A.

Figure 6B
Uncropped Western blots

Myocardin

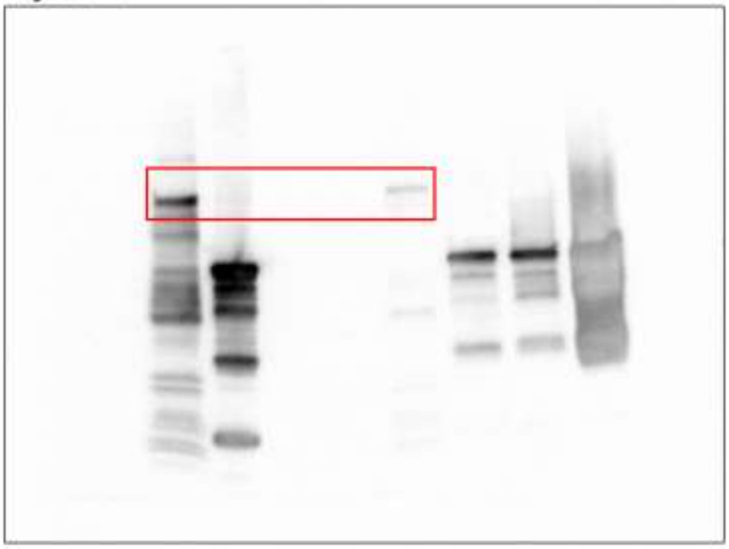

Ponceau S staining

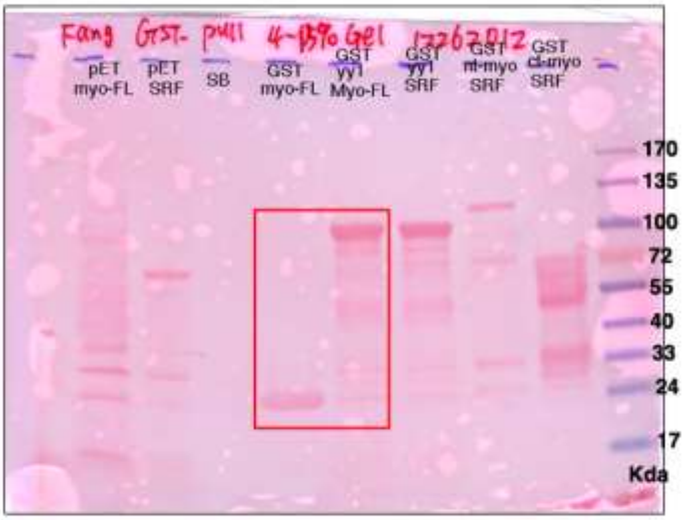

Figure S4B. Uncropped Western blot images for Fig 6B. Boxed bands on the original images by the red lines were cropped for Figure 6B. The information of each lane in the red box is the same as the corresponding lane shown in Figure 6B. All other lanes are not shown in Figure 6B.

Uncropped Western blots
Figure 6C

YY1

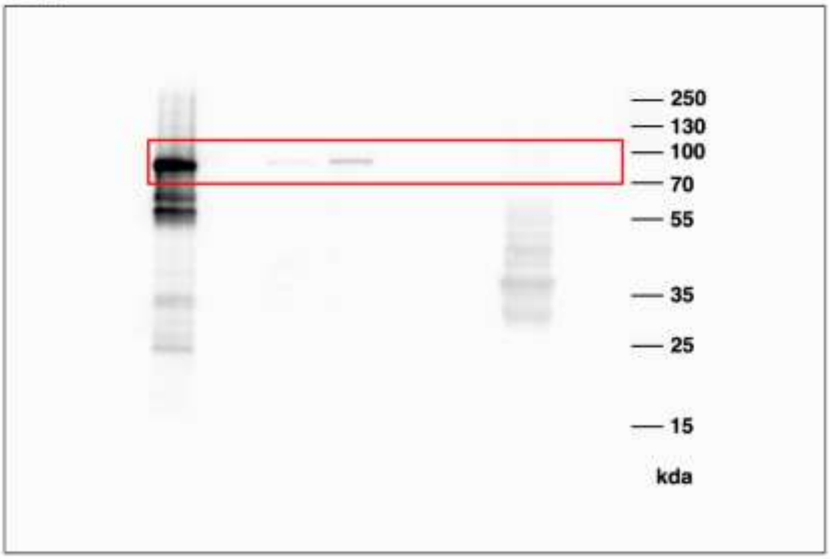

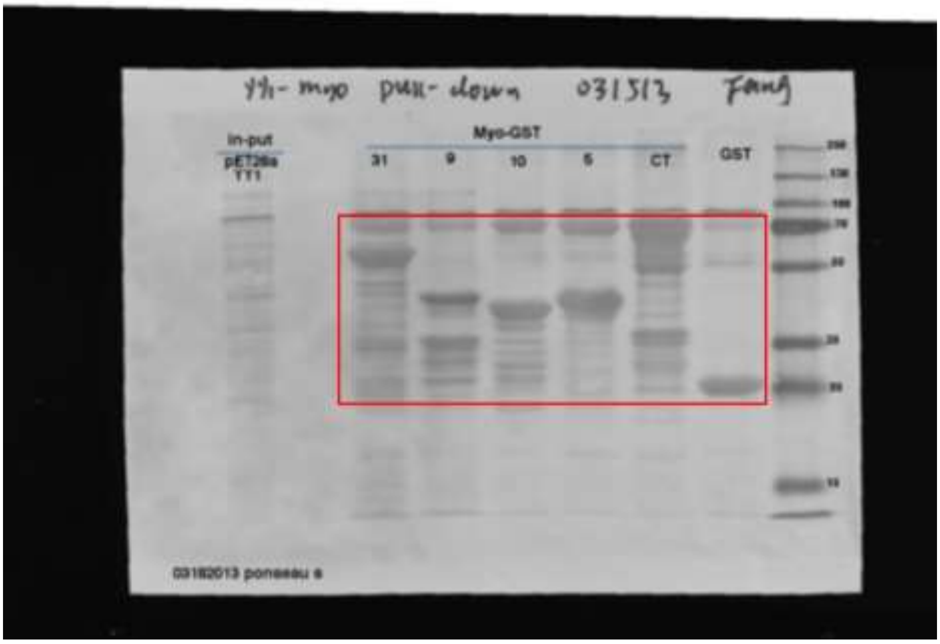

Figure S4C. Uncropped Western blot images for Fig 6C. Boxed bands on the original images by the red lines were cropped for Figure 6C. The information of each lane in the red box is the same as the corresponding lane shown in Figure 6C. All other lanes are not shown in Figure 6C.

Uncropped Western blots
Figure 6D

YY1

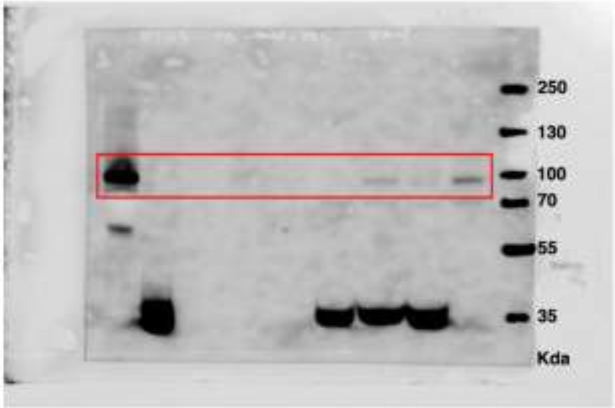

SRF

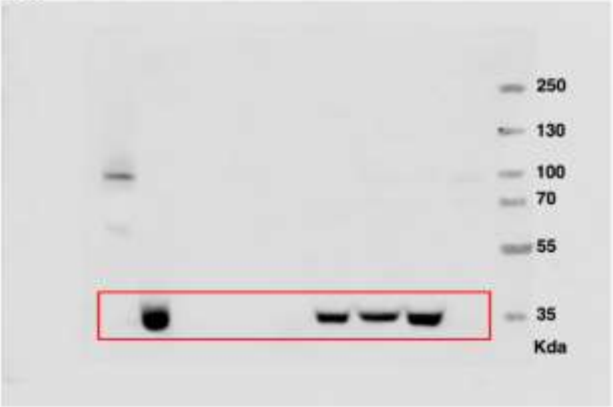

Ponceau S staining

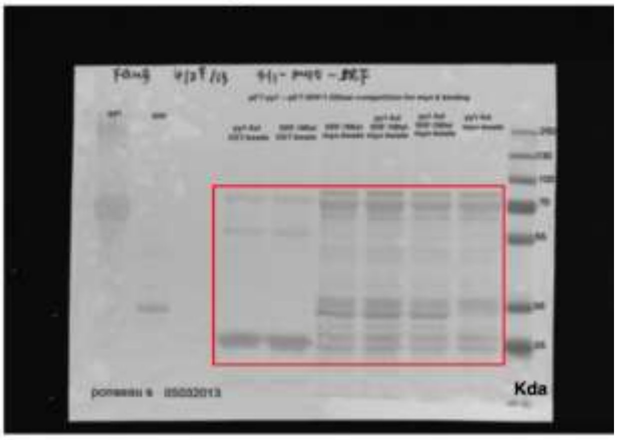

Figure S4D. Uncropped Western blot images for Fig 6D. Boxed bands on the original images by the red lines were cropped for Figure 6D. The information of each lane in the red box is the same as the corresponding lane shown in Figure 6D. All other lanes are not shown in Figure 6D.

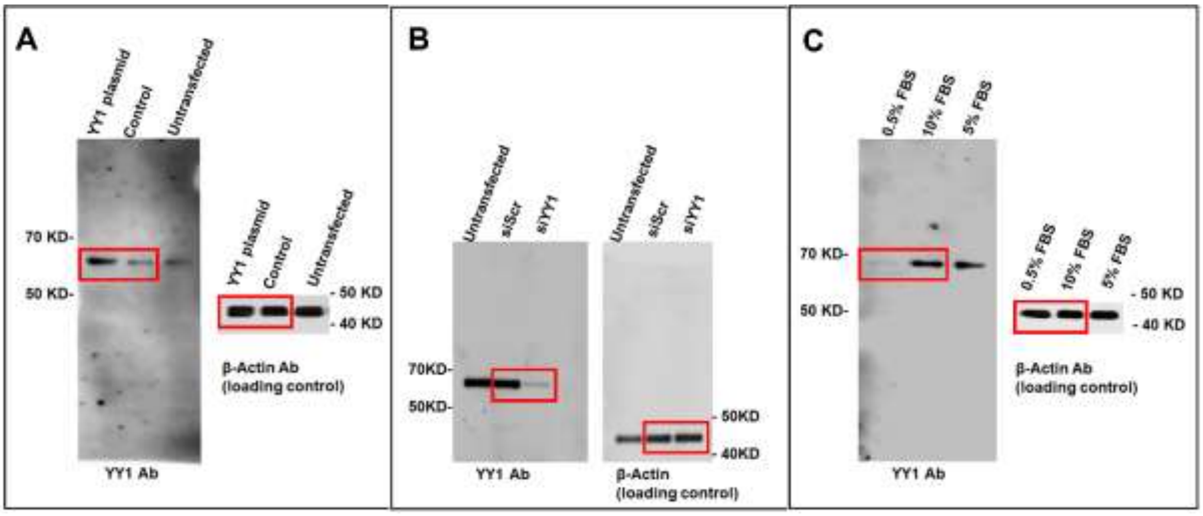

Figure S5. Uncropped original Western blot images. Boxed bands on the original images (A-C) by the red lines were cropped for Figure S2A, S2B and Figure 7A, respectively.
